# Anti-tumor effects of the ethanolic extract of *Trichosanthes kirilowii* seeds in colorectal cancer

**DOI:** 10.1186/s13020-019-0263-8

**Published:** 2019-10-07

**Authors:** Su Mi Park, Sang Kyu Jeon, Ok Hyeon Kim, Jung Yun Ahn, Chang-Hyun Kim, Sun-Dong Park, Ju-Hee Lee

**Affiliations:** 1 Department of Korean Medicine, College of Korean Medicine, Dongguk University, Goyang, 10326 Republic of Korea; 2 Department of Medicine, College of Medicine, Dongguk University, Goyang, 10326 Republic of Korea

**Keywords:** Trichosanthis semen, Colorectal cancer, Anti-cancer, Apoptosis, Anti-tumor

## Abstract

**Background:**

Trichosanthis semen, the seeds of *Trichosanthes kirilowii* Maxim. or *Trichosanthes rosthornii* Harms, has long been used in Korean medicine to loosen bowels and relieve chronic constipation. Although the fruits and radixes of this medicinal herb and their constituents have been reported to exhibit therapeutic effects in various cancers, the anti-cancer effects of its seeds have been relatively less studied. In this study, we investigated the effects of an ethanolic extract of *T. kirilowii* seeds (TKSE) against colorectal cancer and its mechanism.

**Methods:**

The anti-tumor effects of the TKSE were evaluated in HT-29 and CT-26 colorectal cancer cells and in a CT-26 tumor-bearing mouse model.

**Results:**

TKSE suppressed the growth of HT-29 and CT-26 cells (both colorectal cancer cell lines) and the cytotoxic effect of TKSE was greater than that of 5-fluorouracil (5-Fu) in HT-29 cells. TKSE significantly induced mitochondrial membrane potential loss in HT-29 and CT-26 cells and dose-dependently inhibited Bcl-2 expression and induced the cleavages of caspase-3 and PARP. In particular, TKSE at 300 µg/mL induced nuclear condensation and fragmentation in HT-29 cells. Furthermore, TKSE dose-dependently inhibited activations of the Akt/mTOR and ERK pathways, and markedly induced the phosphorylation of AMPK. An AMPKα inhibitor (compound C) effectively blocked the TKSE-induced mitochondrial dysfunction. In addition, TKSE attenuated the hypoxia-inducible factor-1α/vascular endothelial growth factor signaling pathway in HT-29 cells under hypoxic-mimic conditions and inhibited migration and invasion. Oral administration of TKSE (100 or 300 mg/kg) inhibited tumor growth in a mouse CT-26 allograft model but was not as effective as 5-Fu (the positive control), which was administered intraperitoneally. In the same model, 5-Fu caused significant body weight loss, but no such loss was observed in TKSE-treated mice.

**Conclusion:**

Taken together, these results suggest TKSE has potent anti-tumor effects which might be partly due to the activation of AMPK, and the induction mitochondrial-mediated apoptosis in colorectal cancer cells. These findings provide scientific evidence supporting the potential use of TKSE as a complementary and alternative medicine for the treatment of colorectal cancer.

## Background

According to the Global Cancer Statistics published by Bray et al. in 2018, the incidences of cancer and cancer-associated mortalities are rapidly increasing worldwide and colorectal cancer (cancers of the colon, rectum, and anus) ranks third in terms of incidence but second in terms of mortality [1]. Many lifestyle-related factors have been linked to colorectal cancer. In fact, links between diet, alcohol, weight, and exercise and colorectal cancer risk are some of the strongest for any type of cancer [2]. Colorectal cancer can be treated using a number of standard methods, such as surgery, chemotherapy, targeted therapy, radiotherapy, and immunotherapy based on considerations of tumor size and location, cancer stage, whether the cancer is recurrent or not, and general patient health status. Drugs commonly used to treat colorectal cancer include 5-fluorouracil (5-Fu), capecitabine (Xeloda), irinotecan (Camptosar), oxaliplatin (Eloxatin), trifluridine, and trifluridine/tipiracil (Lonsurf), which are used singly or in combination to increase response rates and minimize drug resistance [3]. However, the long-term use of these treatments causes serious side effects and markedly reduces quality of life. Recently, interest in complementary and alternative medicine has increased worldwide due to their efficacies and limited side effects and these approaches are now considered attractive options for the prevention and treatment of colorectal cancer [4].

*Trichosanthes kirilowii* Maxim. and *Trichosanthes rosthornii* Harms (family: Cucurbitaceae) have been and continue to be used extensively in traditional Oriental medicine. The seeds, fruits, pericarps, and roots of these plants are widely used to treat coughs, inflammation, diabetes, and constipation [5]. Since trichosanthis semen, the seeds of *T. kirilowii* or *T. rosthornii*, is a known expectorant with febrifugal properties and aids digestion and excretion, it has long been used in traditional Chinese medicine to treat constipation, inflammation, and coughs [6, 7]. Donguibogam, which is a classical textbook of traditional Korean medicine, states trichosanthis semen reduces sputum production and swelling, and has detoxifying properties [7]. Furthermore, based on Bonchojeonghwa (本草精華), a quintessence of knowledge on science of medicinal ingredients (medicinal phytology/herbal science), trichosanthis semen can be used to relieve hematochezia, dysentery, and leukorrhea [8]. In the scientific literature, trichosanthis semen has been reported to have anti-hepatocarcinogenic, anti-diabetic, anti-inflammatory, and anti-oxidative effects [9–13]. Although the fruits and radixes of *T. kirilowii* and their constituents have shown therapeutic efficacy against various cancers, the anti-cancer effects and action mechanisms of its seeds have not been examined in depth. In this study, we investigated the effects of an ethanolic extract of *T. kirilowii* seeds (TKSE) against colorectal cancer and the mechanisms involved, using colorectal cancer cell lines and a CT-26 tumor-bearing mouse model.

## Materials and methods

### Chemicals and reagents

Fetal bovine serum (FBS), cell culture media, penicillin/streptomycin, and all other reagents used for cell culture studies were purchased from Welgene (Gyeongsan, Korea). 3-(4,5-dimethylthiazol-2-yl)-2,5-diphenyl-tetrazolium bromide (MTT), 4,6-diamidino-2-phenylindole (DAPI), 5-Fu, rhodamine 123 (Rh123), CoCl_2_, compound C, crystal violet solution, and other reagents were purchased from Sigma-Aldrich (St. Louis, MO, USA). Anti-Akt, anti-p-Akt, anti-p-AMPK, anti-Bcl-2, anti-caspase-3, anti-cleaved caspase-3, anti-p-ERK, anti-PARP, anti-mTOR, anti-p-mTOR, anti-p70S6K1, anti-p-p70S6K1, and anti-p-4E-BP1 antibodies were supplied by Cell Signaling Technologies (Danvers, MA, USA). Anti-β-actin and anti-vascular endothelial growth factor (VEGF) antibodies, horseradish peroxidase (HRP)-conjugated goat anti-rabbit, and goat anti-mouse IgGs were purchased from Santa Cruz Biotechnology (Santa Cruz, CA, USA). Anti-hypoxia-inducible factor-1α (HIF-1α) antibody was obtained from BD Biosciences (SanJose, CA, USA). Dimethyl sulfoxide (DMSO) was purchased from AppliChem (Darmstadt, Germany) and Junsei Chemical Co. (Tokyo, Japan).

### Preparation of TKSE

*Trichosanthes kirilowii* (Maximowicz) seeds were purchased as dried herbs from Humanherb (Gyeongsan, Korea), complied with the good manufacturing practices (GMP) guidelines issued by the Korea Food and Drug Administration (KFDA), and were authenticated by Professor Sun-Dong Park (Department of Prescriptions, College of Korean Medicine, Dongguk University). A voucher specimen (No. DUMCKM2015-109) was deposited at the College of Korean Medicine, Dongguk University. Briefly, seeds of *T. kirilowii* (200 g) were coarsely ground and extracted in 70% ethanol (800 mL) by heating at 80 °C for 4 h. Extracts were filtered and concentrated under reduced pressure using a rotary vacuum evaporator (EYELA, Japan). Condensed extracts were lyophilized using a freeze dryer (EYELA) and stored at 4 °C. The yield of dried extract (TKSE) was 4.5% (w/w) of dried herb weight.

### HPLC (high-performance liquid chromatography)

The contents of two standard compounds 3,29-dibenzoyl karounitriol (ChemFaces, Wuhan, China) and cucurbitacin B (Sigma-Aldrich) in TKSE was analyzed using a Thermo Scientific™ UltiMate™ 3000 HPLC system (Thermo Fisher Scientific, Waltham, MA, USA) coupled with UV/VIS diode array detector controlled by Chromeleon 6.8 software. Separations were performed using an INNO C18 column (4.6 mm × 250 mm, 5 µm, Youngjin Biochrom, Korea) at a constant column temperature of 40 °C for apolar molecule analysis (3,29-dibenzoyl karounitriol) and of 30 °C for a polar compound analysis (cucurbitacin B). 3,29-Dibenzoyl karounitriol was separated under isocratic elution with acetonitrile with 0.1% phosphoric acid and 1.0 mL/min flow rate for 20 min. The mobile phases for cucurbitacin B were 0.1% trifluoroacetic acid (A) and acetonitrile (B) and the gradient program was applied as follows: 10% B for 0–25 min, 60% B for 25–30 min, 100% B for 30–35 min, and 10% B for 36–40 min. The flow rate was 0.8 mL/min and an injection volume of 10 μL was used. The detection wavelengths were 230 nm.

### Cell culture

A human colorectal adenocarcinoma cell line (HT-29) and a murine colorectal carcinoma cell line (CT-26) were obtained from the American Type Culture Collection (ATCC, Manassas, VA, USA). HT-29 cells were grown in Roswell Park Memorial Institute Media 1640 (RPMI-1640), and CT-26 cells were cultured in Dulbecco’s modified Eagle’s medium (DMEM) supplemented with 10% FBS and 1% penicillin/streptomycin (Gibco BRL, Gaithersburg, MD, USA). Cultures were maintained at 37 °C in a CO_2_ incubator in a controlled humidified atmosphere composed of 95% air and 5% CO_2_.

### Cell viability assay

Cell viabilities were evaluated using an MTT assay. Briefly, cells were plated at 2–10 × 10^3^ cells per well in 96-well plates and treated with either DMSO (control) or with various concentrations of TKSE (10–500 μg/mL) or 5-Fu (1–50 μM) for 24 h or 48 h. After incubation, viable cells were stained with MTT solution (0.2 mg/mL, 3 h) and the formazan crystals so obtained were dissolved by adding 100 μL DMSO. Absorbances were measured at 540 nm using a multimode microplate reader (Tecan, Research Triangle Park, NC, USA).

### Measurement of mitochondrial membrane potentials

Mitochondrial membrane potentials were measured by flow cytometry using rhodamine 123. HT-29 and CT-26 cells were treated with TKSE (0–500 μg/mL) for 24 h and then stained with 0.05 μg/mL of rhodamine123 for 30 min and harvested by trypsinization. After washing with phosphate buffered saline (PBS) containing 1% FBS, changes in mitochondrial membrane potential were assessed by measuring fluorescence intensities using a CytoFLEX flow cytometer.

### DAPI (4,6-diamidino-2-phenylindole) staining

HT-29 cells were plated onto 18 mm cover glasses in RPMI-1640 at ~ 70% confluence for 24 h. Cells were then treated with 300 μg/mL TKSE for 24 h, fixed in ice-cold 4% para-formaldehyde, and washed with PBS. After mounting cover glasses on glass slides using Fluoroshield™ with DAPI histology mounting medium (Sigma-Aldrich), fluorescence images were captured using an inverted fluorescence microscope (ECLIPSE Ts2-FL; Nikon, Tokyo, Japan) equipped with a monochrome camera (DS-Qi2, Nikon).

### Western blotting

Cells were washed with ice-cold PBS buffer and lysed with radioimmunoprecipitation assay (RIPA) buffer (Thermo Scientific, Rockford, IL, USA), containing protease/phosphatase inhibitor cocktail (GenDEPOT, Barker, TX, USA). Total protein concentrations were quantified using a bicinchoninic acid (BCA) assay kit (Thermo Scientific). Proteins (40 μg) were separated by 8–12% sodium dodecyl sulfate–polyacrylamide gel electrophoresis (SDS-PAGE) and transferred to polyvinylidene fluoride membranes (EMD Millipore, Bedford, MA, USA). Membranes were blocked in 5% skim milk for 3 h at room temperature and incubated overnight at 4 °C with primary antibodies (1:1000 v/v), rinsed, and incubated with secondary antibodies conjugated with horseradish peroxidase (1:3000 v/v) for 1 h at room temperature. After rinsing, protein bands were visualized using an enhanced chemiluminescence system (Amersham Biosciences, Piscataway, NJ, USA).

### ELISA (enzyme-linked immunosorbent assay)

HT-29 cells were seeded in 6-well culture plates and on following days, pre-treated with various concentrations of TKSE (50–500 μg/mL) for 1 h and then exposed to 100 μM CoCl_2_ for 24 h to mimic hypoxic conditions. Amounts of VEGF secreted into media were measured using a human VEGF ELISA kit (AbFrontier, Seoul, Korea) according to the manufacturer’s instructions.

### Scratch wound healing migration assay

HT-29 cells were seeded on 6-well culture plates and wounded with a razor blade at ~ 90% confluence. After removing detached cells by washing with PBS, plates were further incubated in medium supplemented with 5% FBS containing various concentrations of TKSE (50–300 μg/mL). After 48 h, cells were rinsed with PBS and fixed in absolute methanol. Cell migratory behavior was then observed under a phase-contrast microscope (Olympus) and images were digitally captured.

### Matrigel transwell invasion assay

Matrigel transwell invasion assays were performed as previously described [14]. In brief, the upper chambers of a 24-well transwell plate were coated with diluted BD Matrigel™ Basement Membrane Matrix (BD Biosciences, Beit-HaEmek, Israel) and incubated at 37 °C for 3–4 h to cause gelling. HT-29 cells in RPMI-1640 containing 1% FBS and 50–300 μg/mL of TKSE or 0.1% DMSO were added to the upper chamber at a density of 5 × 10^4^ cells/500 μL and lower wells were filled with 750 μL of medium containing 10% FBS as a chemo-attractant. Plates were incubated at 37 °C for 72 h, and non-invading cells on the upper surfaces of transwell inserts were removed with a cotton swab. Cells that travelled to lower surfaces were fixed with absolute methanol, stained with crystal violet, and observed under a light microscope.

### Tumor-bearing mice experiments

Six-week-old male BALB/c mice were purchased from Orient Bio Inc. (Gyeonggi-do, Korea) and maintained in a pathogen-free facility (23 ± 2 °C, 50 ± 15% relative humidity, under a 12:12 h light/dark cycle) with free access to food and water. Animal experiments were approved beforehand by the Institutional Animal Care and Use Committee of Dongguk University (Approval No. IACUC-2018-011). After acclimation for 1 week, 1 × 10^6^ CT-26 cells in 100 μL of PBS were subcutaneously inoculated into right flanks. When tumor volumes reached ~ 50 mm^3^, mice were randomly distributed based on tumor volume and body weight into four groups (n = 5/group) as follows: Control group (water as a vehicle), TKSE (L) group (low dose, 100 mg/kg), TKSE (H) group (high dose, 300 mg/kg), and 5-Fu group (50 mg/kg). TKSE was orally administrated five times per week for 3 weeks and 5-Fu was intraperitoneally administrated twice per week for 3 weeks. Body weights and tumor sizes were recorded twice and three times per week, respectively. Tumor volumes were calculated using 0.5 × long axis × (short axis)^2^. At the end of treatment, mice were sacrificed and blood was collected by cardiac puncture. The tumors were excised from the mice and weighed. Serum samples were isolated from blood and stored at − 80 °C. Serum glutamic oxaloacetic transaminase (GOT) and glutamic pyruvic transaminase (GPT) levels were detected using a commercial assay kits (Asan Pharm., Republic of Korea).

### Statistical analysis

Results are expressed as the means ± standard deviations (SD; for in vitro data) or the mean ± standard error of the mean (SEM; for in vivo data). Statistical significance was determined by one-way ANOVA followed by Tukey’s multiple comparison test using GraphPad Prism software (GraphPad Software Inc., San Diego, CA, USA). Differences were considered statistically significant when *p* values were < 0.05.

## Results

### TKSE inhibited growth of colorectal cancer cells

**Fig. 1:**
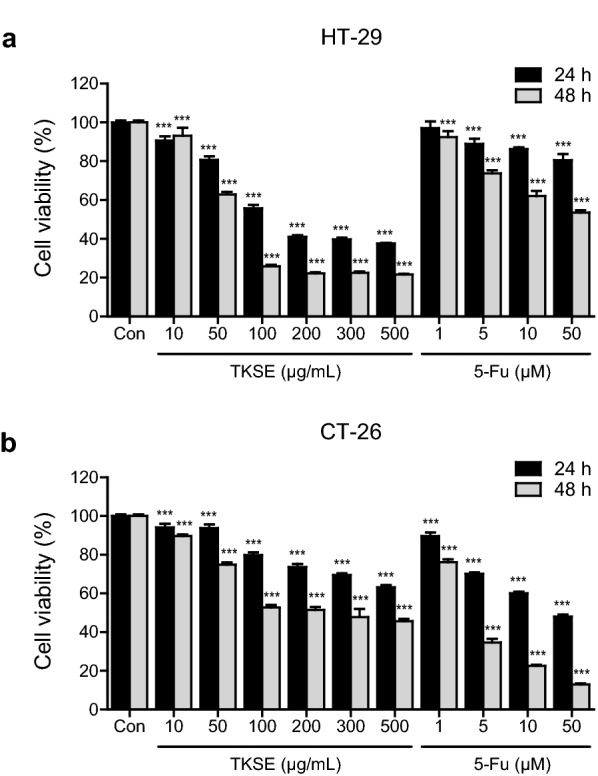
Effect of TKSE on the growth of colorectal cancer cells. The inhibitory effects of TKSE on the proliferations of HT-29 (**a**) and CT-26 (**b**) cells were assessed using an MTT assay. 5-Fluorouracil (5-FU) was used as a positive control. Results are expressed as percentage cell proliferations relative to vehicle-treated controls. Results are presented as mean ± SDs. Significance vs. vehicle-treated controls, ***p < 0.001

To investigate the effect of TKSE on the growth of colorectal cancer cells, we first performed MTT cell viability assays. HT-29 and CT-26 cells were treated with various concentrations of TKSE (10–500 μg/mL) or 5-Fu (1–50 μM; positive control) for 24 and 48 h. Treatment with TKSE decreased the viabilities of HT-29 and CT-26 cells in a dose- and time-dependent manner as compared with vehicle-treated controls (Fig. 1a, b). At 48 h, the IC_50_ values of TKSE were 67.3 and 239.1 µg/mL in HT-29 and CT-26 cells, respectively, which implied TKSE had a greater effect on HT-29 cells. Notably, the cytotoxic effect of TKSE on HT-29 human colorectal cancer cells was also greater than that of 5-Fu.

### TKSE caused mitochondrial dysfunction in colorectal cancer cells

**Fig. 2:**
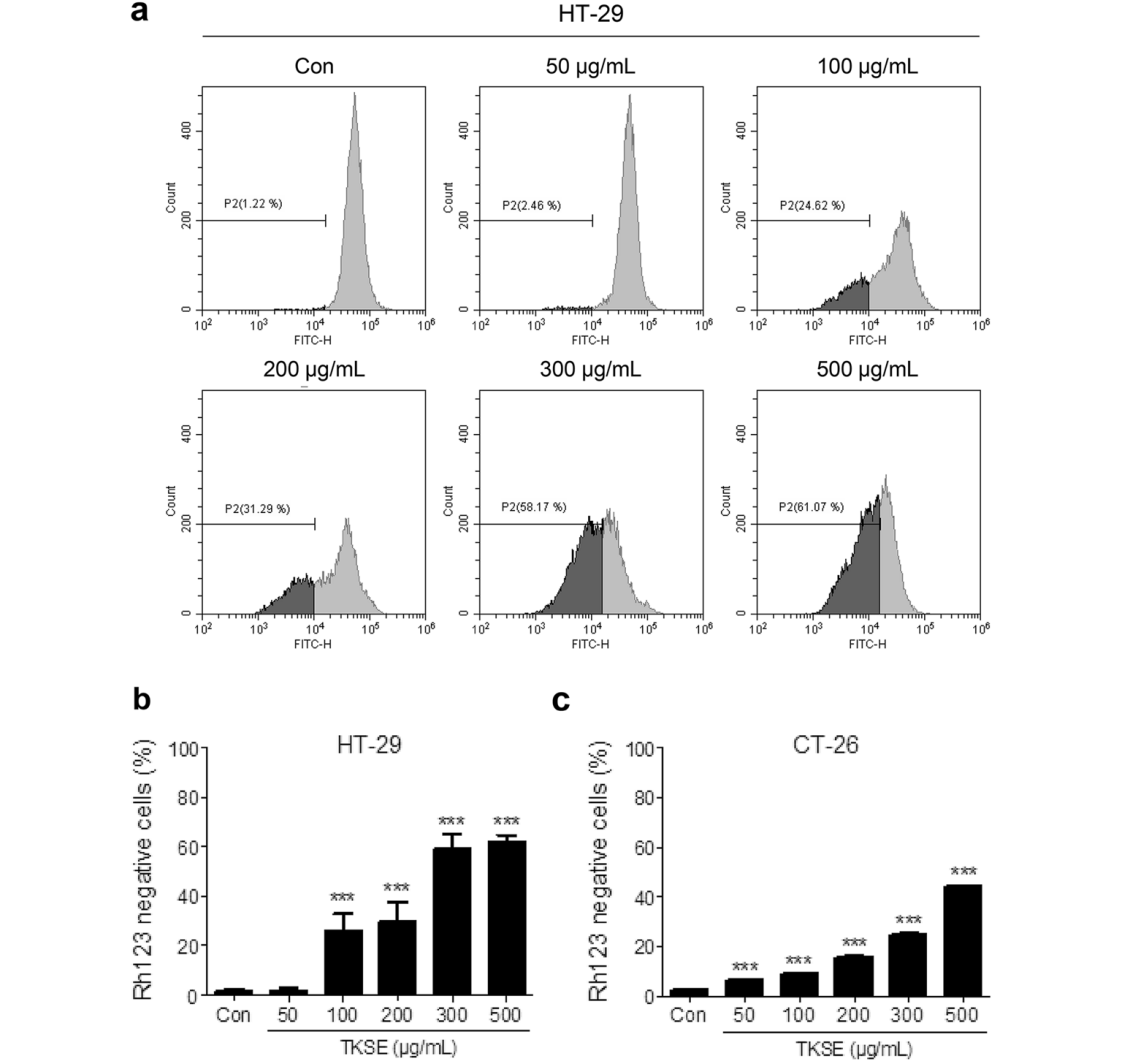
Effect of TKSE on mitochondrial dysfunction in colorectal cancer cells. **a** Mitochondrial damage caused by TKSE (50–500 µg/mL) was investigated using rhodamine 123. The left side of the fluorocytogram indicates the percentage of cells showing loss of mitochondrial membrane potential. **b** Percentage of mitochondrial membrane potential losses in the vehicle-treated controls and TKSE-treated HT-29 cells. **c** Percentage of mitochondrial membrane potential losses in the vehicle-treated controls and TKSE-treated CT-26 cells. Significance vs. vehicle-treated controls, ***p < 0.001. Rh123; rhodamine 123

To determine whether the inhibitory effect of TKSE on cell growth was related to mitochondrial dysfunction, we measured mitochondrial membrane potential using flow cytometry after staining cells with rhodamine 123. HT-29 and CT-26 cells were treated with different concentrations of TKSE (50–500 µg/mL) for 24 h and then stained with rhodamine 123 dye for 30 min. Flow cytometer determined fluorescence intensity measurements showed that TKSE dose-dependently increased the proportion of rhodamine 123-negative cells, indicating loss of mitochondrial membrane potential in both HT-29 and CT-26 cells, though this loss was greater in HT-29 cells than in CT-26 cells (Fig. 2). Consistent with our cell viability assay results, TKSE at 300 and 500 µg/mL resulted in largest mitochondrial membrane potential losses (59.0 ± 6.0 and 62.1 ± 2.5%, respectively) in HT-29 cells (Fig. 2a, b), and thus, TKSE was administered at concentration up to 300 µg/mL to these cells in the following experiments.

### TKSE induced apoptosis in colorectal cancer cells

**Fig. 3:**
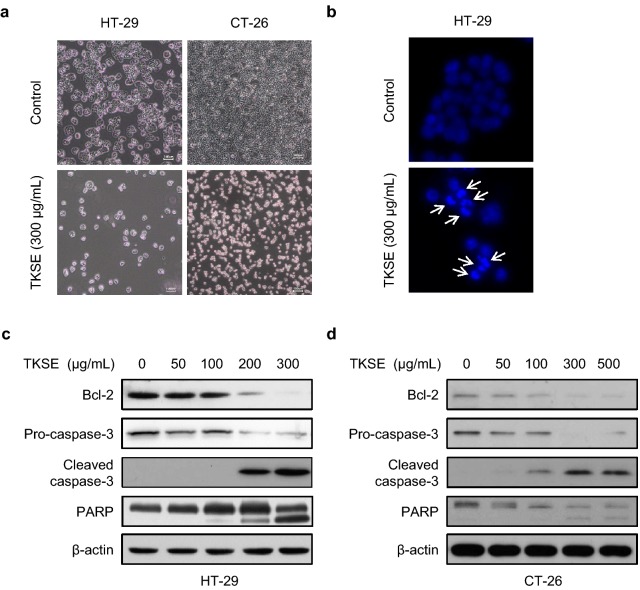
Effect of TKSE on colorectal cancer cell apoptosis. **a** HT-29 and CT-26 cells were treated with 300 µg/mL TKSE for 24 h. Representative photographic images are shown (Original magnification ×100). Scale bar = 100 µm. **b** The induction of apoptosis by TKSE (300 µg/mL) was assessed by DAPI staining (×100). The expressions of apoptosis-related proteins were assayed by western blotting HT-29 (**c**) or CT-26 (**d**) cells treated with TKSE at the indicated doses for 24 h

Morphological changes in HT-29 and CT-26 cells treated with 300 µg/mL TKSE for 24 h were observed using an inverted microscope. As shown in Fig. 3a, treatment with TKSE was associated with inhibited cell growth, cell shrinkage, and cell detachment from culture plates for both HT-29 and CT-26 cells. To determine whether growth inhibition and loss of mitochondrial membrane potential by TKSE was linked with apoptosis, cell nuclei were observed under a fluorescence microscope. HT-29 cells were incubated with 300 µg/mL TKSE for 24 h and stained with DAPI for microscopic observations. As was expected, nuclear condensation and fragmentation (the morphological hallmarks of apoptosis) were observed in HT-29 cells treated with TKSE (Fig. 3b). In addition, TKSE dose-dependently decreased the expression of anti-apoptotic Bcl-2 and increased cleavages of caspase-3 and PARP in HT-29 and CT-26 cells (Fig. 3c, d).

### TKSE affected Akt, ERK, and AMPK signaling pathways in HT-29 cells

**Fig. 4:**
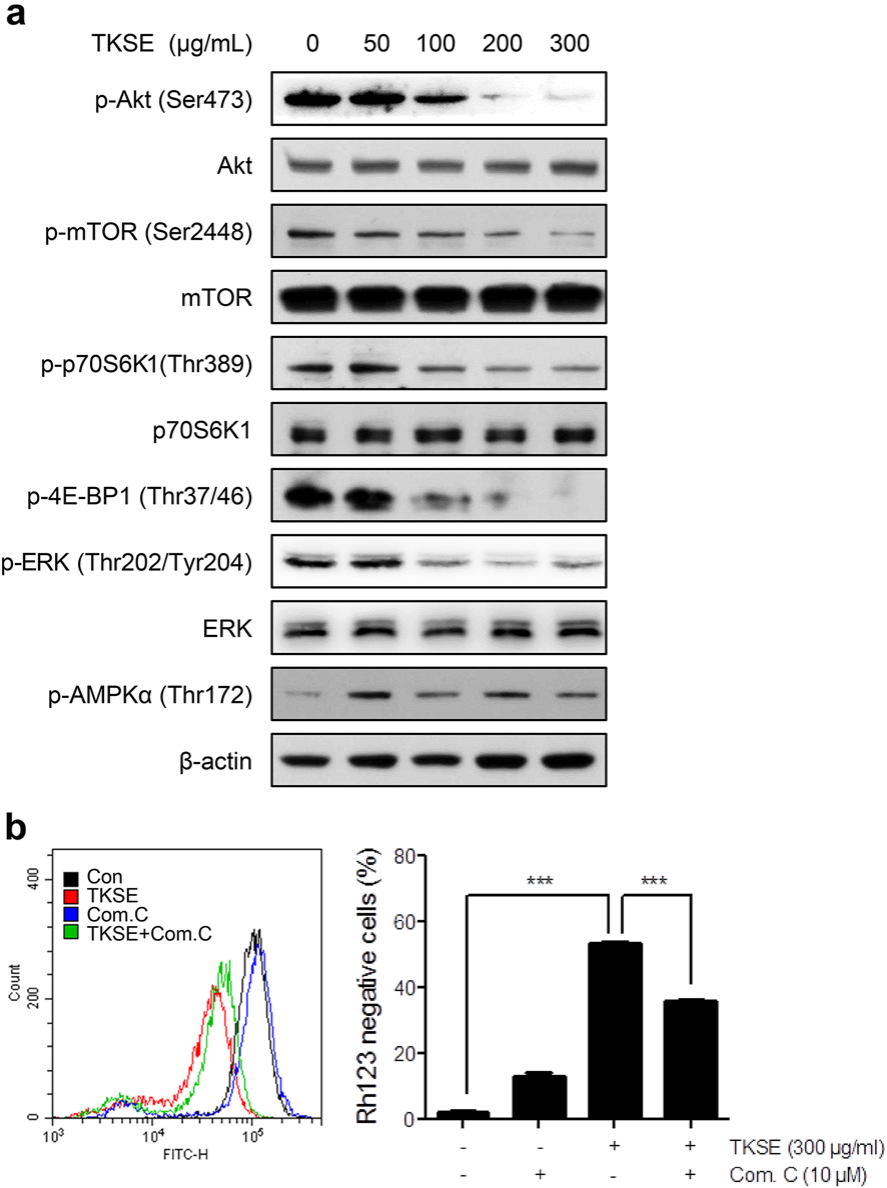
Effects of TKSE on Akt, ERK, and AMPK signaling in HT-29 cells. **a** HT-29 cells were treated with the indicated concentrations of TKSE for 6 h, and the protein levels of Akt and its downstream proteins (mTOR, p70S6K1, and 4E-BP1), ERK, AMPK, and their phosphorylated forms were analyzed by western blot. **b** Suppression of the effect of TKSE on mitochondrial membrane potential by compound C. ***p < 0.001

To investigate the molecular mechanisms responsible for TKSE-induced cell growth inhibition, we determined the protein levels of proliferation-related signaling pathways, that is, those of Akt, ERK, and AMPK. In HT-29 cells treated with different concentrations of TKSE (50–300 µg/mL) for 6 h, TKSE dose-dependently reduced the expressions of p-Akt, p-mTOR, p-p70S6K1, p-4E-BP1, and p-ERK, without affecting their total levels (Fig. 4). We also analyzed whether the inhibitory effects of TKSE on mTOR signaling were associated with AMPK activation. As shown in Fig. 4a, TKSE treatment of HT-29 cells increased the phosphorylation of AMPK at Thr172 (a surrogate of AMPK activity) from a concentration of 50 µg/mL TKSE. To examine the relationship between the AMPK activation by TKSE treatment and the inhibitory effect of colorectal cancer cells, we measured mitochondrial membrane potential after pre-treating with compound C as an AMPK inhibitor for pharmacological inhibition in HT-29 cells. As expected, the ability of TKSE (300 µg/mL) to induce mitochondrial membrane potential loss was significantly suppressed by compound C (10 µM) pre-treatment (Fig. 4b). These results suggest the inhibitory effect of TKSE on the growth of colorectal cancer cells was mediated through the down-regulation of proliferation-related signaling pathways.

### TKSE suppressed the metastatic abilities of HT-29 cells

**Fig. 5:**
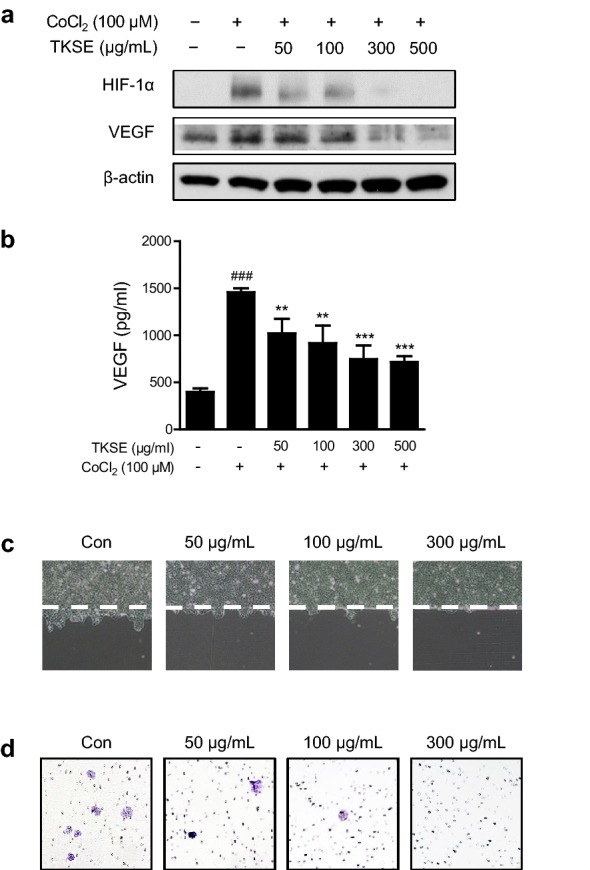
Effects of TKSE on the malignant properties of HT-29 cells. **a** The expressions of HIF-1α and its downstream gene VEGF were assessed by western blotting in hypoxia-induced HT-29 cells treated with TKSE. **b** Production of VEGF in hypoxia-induced HT-29 cells treated with TKSE. Cells were treated with various concentrations of TKSE under hypoxia for 24 h. Results are presented as the means ± SDs of triplicate wells. Significance vs. CoCl_2_-treated group, **p < 0.01, ***p < 0.001. **c** A wound healing migration assay was used to observe the effects of TKSE on HT-29 cell motility. **d** Representative images of Matrigel transwell invasion by TKSE-treated HT-29 cells. Original magnification, ×100

Approximately 25% of patients with colorectal cancer have metastatic disease at time of diagnosis, and 50–60% of colorectal cancer patients develop metastasis [15, 16], which is the main cause of cancer-related mortality. Hypoxia is the most common characteristic of solid tumors and drives cancer metastasis by increasing the expression of HIF-1α [17]. To determine whether TKSE affects HIF-1α protein levels, HT-29 cells were treated with various concentrations of TKSE (50–500 μg/mL) under hypoxic-mimicking conditions induced by treating cells with 100 μM CoCl_2_ for 24 h. As shown in Fig. 5a, CoCl_2_ markedly induced HIF-1α expression and TKSE dose-dependently inhibited the induction. Since HIF-1α and its downstream gene VEGF are main regulators of angiogenesis, we also examined the effect of TKSE on the production of VEGF using western blot and ELISA. As was expected, VEGF levels were elevated in hypoxic HT-29 cells, and TKSE at 300 and 500 μg/mL suppressed VEGF levels to lower than control levels (Fig. 5a). In addition, we confirmed that the hypoxic condition induced a 3.66-fold increases in VEGF secretion versus the control (Control, 398.33 ± 37.86 pg/mL; CoCl_2_, 1458.33 ± 40.41 pg/mL), and that TKSE treatment significantly reduced VEGF levels at all concentrations tested (Fig. 5b). To investigate the effect of TKSE on metastasis, we performed cell migration and invasion assays to assess the initial steps of the metastatic process. Results of wound healing migration assays showed that treatment with TKSE for 48 h effectively and dose-dependently inhibited HT-29 cell migration as compared with controls (Fig. 5c). Although slight cytotoxicity was observed at the highest concentration (300 μg/mL) of TKSE in the transwell invasion assays, TKSE markedly suppressed the invasiveness toward the FBS-attractant gradient in the dose-dependent manner (Fig. 5d). These results suggest TKSE suppresses the metastatic abilities of colorectal cancer cells.

### TKSE attenuated tumor growth in CT-26 tumor-bearing mice

**Fig. 6:**
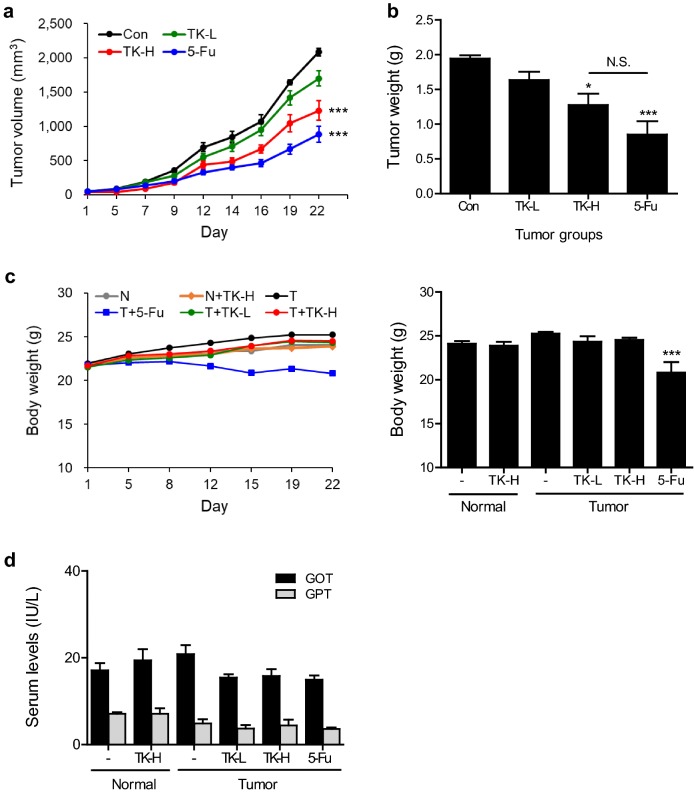
Effect of TKSE on tumor growth in the CT-26 tumor-bearing mouse model. CT-26 cells (1 × 10^6^) were subcutaneously inoculated into the right flanks of BALB/c mice. When tumor volumes reached ~ 50 mm^3^, TKSE (100 or 300 mg/kg) was orally administered five times per week for 3 weeks and 5-Fu (50 mg/kg) was intraperitoneally administered twice per week for 3 weeks. Body weights and tumor sizes were recorded twice and three times per week, respectively. **a** Tumor growth curve of mice treated with vehicle (Con), 100 mg/kg TKSE (TK-L), 300 mg/kg TKSE (TK-H), or 50 mg/kg 5-Fu (5-Fu) during treatment. **b** Tumor weights. **c** Body weight changes of mice in each experimental group. **d** Serum levels of the liver-specific enzymes GOT and GPT. N; normal group, T; tumor groups. *NS* not significant. Results are presented as means ± SEMs (n = 5). Significance *vs.* the vehicle-treated tumor-bearing controls, *p < 0.05, ***p < 0.001

To determine whether TKSE inhibits tumor growth in vivo, we established an allograft colon carcinoma animal model by implanting BALB/c mice with CT-26 cells. TKSE was orally administrated 5 times per week at 100 or 300 mg/kg from when tumors reached an average volume of 50 mm^3^. TKSE administration at both doses for 3 weeks significantly suppressed tumor growth as compared with vehicle-treated tumor controls but was not as effective as 5-Fu (the positive control), which was administered intraperitoneally (Fig. 6a). Average tumor volumes of 100 or 300 mg/kg TKSE-treated mice were about 18.4% and 41% lower than those of vehicle-treated controls, respectively (Fig. 6a). In addition, the tumor weights of mice treated with 100 or 300 mg/kg of TKSE also were reduced by about 15.8% and 34.4%, respectively, versus vehicle-treated controls (Fig. 6b). To assess general toxicity, we measured the body weights of tumor-bearing mice. TKSE treated mice showed no significant change in body weight versus treatment naïve normal controls, suggesting no toxicity under our test conditions (Fig. 6c). However, 5-Fu treated mice showed significant body weight losses as compared with treatment naïve normal or vehicle-treated tumor controls (normal vs. 5-Fu; 14% reduction, tumor controls vs. 5-Fu; 18% reduction). In addition, we measured GOT and GPT in serum of experimental mice and found that there was no statistically significant difference between treatment groups and treatment naïve normal controls, suggesting no hepatic toxicity (Fig. 6d).

### HPLC analysis

**Fig. 7:**
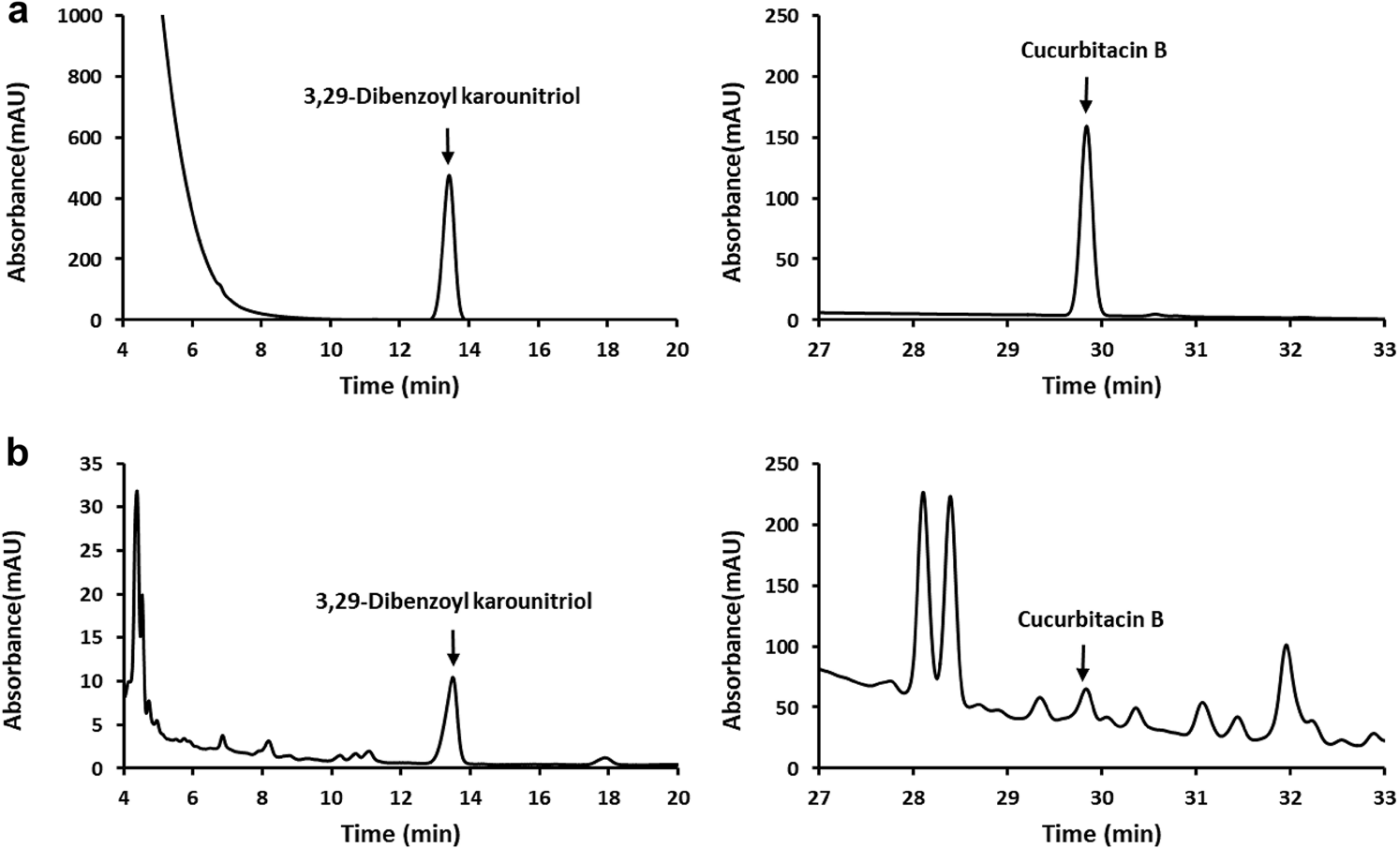
HPLC chromatograms of TKSE. **a** Chromatograms of standard compounds, 3,29-dibenzoyl karounitriol (left panel) and cucurbitacin B (right panel). **b** Chromatograms of TKSE

For qualitative HPLC analysis, we selected 3,29-dibenzoyl karounitriol and cucurbitacin B as controls of trichosanthis semen. Figure 7 shows chromatograms of TKSE and two standard compounds. Compared the TKSE with the standard chromatograms, the peaks at retention times 13.350 and 29.827 min were similar to the 3,29-dibenzoyl karounitriol and cucurbitacin B, respectively.

## Discussion

Colorectal cancer is one of the most common malignant tumors and is known to metastasize predominantly to liver, lung, and lymph nodes [2]. In the case of advanced colorectal cancer, surgery, chemotherapy, and radiotherapy are using in combination with the goals of prolonging life, alleviating pain, and improving the quality of life. However, the various side effects associated with these treatments significantly reduce quality of life. Herbal medicines containing multiple compounds that interact with multiple targets are regarded as promising candidates for cancer prevention and treatment due to their efficacies and relatively low toxicities.

The plants *T. kirilowii* and *T. rosthornii* have been used extensively for centuries in traditional Oriental medicine, and their roots, fruits, and seeds are accredited with various biological activities that include expectorant activity, anti-inflammatory activity, antioxidant activity, and cytotoxic effects [18]. Several studies have investigated the anti-cancer effects of extracts of other parts of these plants and their constituents, though few have been conducted in vivo. For example, *T. kirilowii* tuber ethanol extract and cucurbitacin D (an active component in this extract) have been reported to inhibit cell growth and induce apoptosis by inhibiting STAT3 activity in breast cancer cells [19], and isoaurone, cucurbitacin B, and 6-(3-hydroxy-4-methoxystyryl)-4-methoxy-2Hpyran-2-one isolated from the methanol extract of *T. kirilowii* seeds were reported to potently inhibit HIF-1 and NF-κB activities [5]. In another study, Minh et al. found eight compounds from the roots of *T. kirilowii* had cytotoxic effects against lung, colon, ovarian, and breast cancer cells [20].

As traditional Korean medicine incorporates several herbs simultaneously in prescriptions, studying the efficacy and mechanism of each herbal source is required and plays a major role in developing new prescriptions. Although the beneficial effects of several important bioactive components present in *T. kirilowii* have been reported, scientific evidence regarding the efficacy and mechanism of action of whole extract of *T. kirilowii* seeds is lacking. Thus, in the present study, we investigated the anticancer effect of TKSE against colorectal cancer and explored the molecular mechanisms responsible with focus on Akt/mTOR, ERK, and AMPK. We report the following novel findings: (1) TKSE inhibited the proliferation of colorectal cancer cells by inhibiting the Akt/mTOR and ERK signaling pathways and activating AMPK, thus inducing apoptosis, (2) TKSE inhibited the metastatic abilities of colorectal cancer cells through the HIF-1α/VEGF pathway, and (3) TKSE exerted the anti-tumor effect without causing any observed toxic effects in our CT-26 tumor-bearing mouse model.

Proliferation and survival related signaling pathways such as PI3K/AKT/mTOR and RAS/RAF/MEK/ERK play important roles in pathogeneses of solid tumors [21], and KRAS, BRAF, and PIK3CA (PI3K) mutations are the most common in colorectal cancer [22]. Therefore, concurrent inhibition of these pathways by combined with MAPK and PI3K pathway inhibitors may be a promising strategy for optimal therapeutic activity in colorectal cancer patients [23]. In fact, Pitts et al. reported enhanced anti-tumor activities for the dual PI3K/mTOR inhibitor PF-502 and the MEK inhibitor PD-901 when used in combination in in vitro and in vivo models of colorectal cancer [24]. Our results show that TKSE strongly downregulated phosphorylations of ERK, Akt, and mTOR and its downstream effectors (p70S6K1 and 4E-BP1).

AMPK is a major regulator of metabolic pathways and has been considered an important target for cancer prevention and treatment because it can inhibit colorectal carcinogenesis and induce the apoptosis of various cancer cells [25]. Oral administration of quercetin reduced tumor volume and activated AMPK in HT-29 tumor xenograft mice [26], and berberine activated AMPK, decreased mTOR (a downstream target of AMPK) levels, and phosphorylated p53 in colorectal cancer cells [27]. In another study, aspirin inhibited mTOR signaling, activated AMPK, and induced autophagy in colorectal cancer cells [28]. Consistent with these observations, we observed TKSE activated AMPK and decreased mTOR activity in HT-29 cells. Furthermore, TKSE inhibited colorectal cancer cell proliferation by regulating these signaling pathways, disrupting mitochondrial membrane potentials, and inducing apoptosis. Our results are consistent with those of an in vitro study, in which TKP, a serine protease purified from *T. kirilowii* fruit, was determined to induce apoptosis via a PI3K/AKT-mediated mitochondria-dependent pathway in human colorectal adenocarcinoma cells [29].

In addition, the present study shows TKSE suppresses the metastatic potential of colorectal cancer cells. As tumors grow, hypoxia develops as a result of uncontrolled rapid cell proliferation and nutrient supply becomes inadequate, and these deficiencies activate various intracellular signaling pathways including the major HIF-1/VEGF pathway and the PI3K/AKT/mTOR, ERK, and NF-ĸB pathways [30]. Furthermore, hypoxia amplifies migratory and invasive properties and increases metastatic potential [30]. HIF-1α is an essential mediator of cellular response to hypoxia and VEGF is a potent angiogenic factor, both are overexpressed in colorectal cancer and prognostic indicators of poor outcome [31]. In the present study, TKSE inhibited HIF-1α protein expression and this diminished intracellular VEGF protein expression and its secretion and reduced the ability of HT-29 cells to migrate and invade.

TKSE was also observed to have anti-tumor effects in vivo. Oral administration of TKSE at 100 or 300 mg/kg dose-dependently suppressed tumor growth in our mouse CT-26 allograft model, though it was not as effective as intraperitoneally administered 5-Fu. However, if tumor bearing mice had been administered TKSE at higher doses than 300 mg/kg or administered via a more direct route, better results might have been obtained. On the other hand, 5-Fu significantly reduced body weights indicating general toxicity, whereas TKSE did not. These results suggest the anti-tumor effect of TKSE is not associated with toxic side effects. Although 5-Fu is one of the most effective chemotherapeutics for the treatment of colorectal cancer, it frequently causes intestinal mucosal injury, and thus, abdominal pain and diarrhea [32, 33]. Moreover, because 5-Fu does not specifically target tumor tissues effectively, many studies have investigated its effects in combination with other anticancer drugs [34]. We suggest further studies be undertaken on the combined use of TKSE and 5-Fu in colorectal cancer. Seeds of *T. kirilowii* have been frequently prescribed as a remedy in traditional Korean medicine for various diseases at a dose of 3–15 g and has been used in higher doses, up to 20 g to treat constipation [35–37]. Though we found that oral administration of 300 mg/kg TKSE potently has anti-tumor effects in the current animal experiments, the relevant human dose can be obtained by the more detailed animal scale-up study at a later date.

3,29-Dibenzoyl karounitriol is used to control the quality of trichosanthis semen in Chinese Pharmacopoeia 2015 [38, 39]. Cucurbitacin B has been found in many Cucurbitaceae plant species and also has been reported to be present in the seeds, fruits, and roots of *T. kirilowii* [39]. In the present study, 3,29-dibenzoyl karounitriol and cucurbitacin B were identified as markers for quality control purpose in the TKSE by HPLC. Many studies have reported that the cucurbitacins possess anti-cancer effects against various cancers [39]. It appears the observed anticancer activity by TKSE in colorectal cancer cells may have been the result of the combined effects of its several active components. To confirm these findings, studies on fractions or single compounds of the trichosanthis semen are needed in the future.

## Conclusions

Our results suggest TKSE has potent anti-tumor effects, and that these effects might be due to the targeting of Akt/mTOR, ERK, and AMPK, induction of mitochondrial-mediated apoptosis, and suppression of colorectal cancer cell metastasis. These findings provide scientific evidence supporting the potential use of TKSE as a complementary alternative medicine for the treatment of colorectal cancer and may be used as a foundation for developing new prescriptions in the future.

## Data Availability

The datasets used and/or analysed during the current study are available from the corresponding author on reasonable request.

## Abbreviations

5-Fu: 5-fluorouracil
ANOVA: analysis of variance
DAPI: 4,6-diamidino-2-phenylindole
DMEM: Dulbecco’s modified Eagle’s medium
DMSO: dimethyl sulfoxide
ELISA: enzyme-linked immunosorbent assay
FBS: fetal bovine serum
GOT: glutamic oxaloacetic transaminase
GPT: glutamic pyruvic transaminase
HIF-1α: hypoxia-inducible factor-1α
HRP: horseradish peroxidase
MTT: 3-(4,5-dimethylthiazol-2-yl)-2,5-diphenyl-tetrazolium bromide
PBS: phosphate-buffered saline
RPMI: Roswell Park Memorial Institute Media
SD: standard deviations
SEM: standard error of the mean
TKSE: ethanolic extract of *Trichosanthes kirilowii* seeds
VEGF: vascular endothelial growth factor
